# Isolation and Identification of Inter-Species *Enterovirus* Recombinant Genomes

**DOI:** 10.3390/v13122390

**Published:** 2021-11-29

**Authors:** Kirsten Bentley, Han Kang Tee, Ashley Pearson, Kym Lowry, Sheila Waugh, Siân Jones, Yoke Fun Chan, David J. Evans

**Affiliations:** 1 Biomedical Sciences Research Complex, School of Biology, University of St. Andrews, St. Andrews KY16 9ST, UK; 2 Department of Medical Microbiology, Faculty of Medicine, University of Malaya, Kuala Lumpur 50603, Malaysia; 3 Centre for Clinical Research, The University of Queensland, Herston, QLD 4029, Australia; 4 Microbiology and Virology, Freeman Hospital, The Newcastle upon Tyne Hospitals NHS Foundation Trust, Newcastle upon Tyne NE7 7DN, UK

**Keywords:** recombination, enterovirus, virus evolution

## Abstract

Positive-strand RNA virus evolution is partly attributed to the process of recombination. Although common between closely genetically related viruses, such as within species of the *Enterovirus* genus of the *Picornaviridae* family, inter-species recombination is rarely observed in nature. Recent studies have shown recombination is a ubiquitous process, resulting in a wide range of recombinant genomes and progeny viruses. While not all recombinant genomes yield infectious progeny virus, their existence and continued evolution during replication have critical implications for the evolution of the virus population. In this study, we utilised an in vitro recombination assay to demonstrate inter-species recombination events between viruses from four enterovirus species, A-D. We show that inter-species recombinant genomes are generated in vitro with polymerase template-switching events occurring within the virus polyprotein coding region. However, these genomes did not yield infectious progeny virus. Analysis and attempted recovery of a constructed recombinant cDNA revealed a restriction in positive-strand but not negative-strand RNA synthesis, indicating a significant block in replication. This study demonstrates the propensity for inter-species recombination at the genome level but suggests that significant sequence plasticity would be required in order to overcome blocks in the virus life cycle and allow for the production of infectious viruses.

## 1. Introduction

Recombination is a common process amongst positive-strand RNA viruses and is a strong driver of virus evolution through the exchange of genomic sequences that can be directly advantageous or result in the removal of deleterious mutations [1,2,3]. The mechanism of replicative recombination requires a strand transfer event in which the template copied by the RNA-dependent RNA polymerase (RdRp) changes during negative-strand synthesis [4]. We have shown recently that this strand transfer event is a ubiquitous and continuous process, resulting in the generation of a wide range of both viable and non-viable genomes [5].

The *Enterovirus* genera are the largest of the *Picornaviridae* family, consisting of 15 species—*Enterovirus* A-L and *Rhinovirus* A-C—and over 300 characterised viruses [6]. All enteroviruses have a single-stranded, positive-sense RNA genome, of approximately 7.5 Kb, encoding a single polyprotein flanked by 5′ and 3′ untranslated regions (UTR) containing the signals for translation and replication. The polyprotein is defined by three gene regions—P1, P2, and P3—and is co- and post-translationally cleaved to yield the structural proteins VP4, VP2, VP3, VP1 from P1, and the non-structural proteins 2A^pro^, 2B, 2C and 3A, 3B^VPg^, 3C^pro^, and 3D^pol^ from P2 and P3, respectively. Poliovirus (PV), the prototype *Enterovirus C* species, is a well-established tool for the study of recombination. The virus undergoes rapid recombination between serotypes 1 and 3 (PV1 and PV3) in recipients of the live-attenuated oral vaccine [7,8], as well as intra-species recombination with closely related co-circulating species C enteroviruses [9,10]. There is evidence to suggest that inter-species recombination between members of the *Enterovirus* genera can occur [11,12], and indeed has occurred [13], although such examples are restricted to the exchange of the 5’ UTR, a functionally constrained region that exhibits significant sequence plasticity [14,15].

As recombinants are relatively rare, when compared with the non-recombinant progeny of parental viruses, studying the process requires the ability to preferentially isolate recombinants. To this end, we developed a poliovirus-based in vitro recombination assay—the CRE-REP assay—that only allows the recovery of recombinant progeny viruses [3,16,17,18]. The assay involves the co-transfection of two RNA templates—acceptor and donor—that are independently unable to produce infectious progeny virus. The acceptor template is a full-length genome containing mutations within a *cis*-acting RNA structure in the 2C coding region [19] that renders the resulting CRE mutant genome able to undergo negative-strand but not positive-strand synthesis [20]. The donor template replicons are modified by replacement of the structural protein-coding region with a luciferase reporter gene allowing replication but that are consequently unable to produce infectious virions. When co-transfected into permissive cells, polymerase-mediated strand transfer events that occur between sequences 5′ to the donor strand 2C defect and sequences 3′ to the acceptor capsid coding region deletion may result in the generation of a recombinant virus that can be isolated for further analysis (Figure S1). The CRE-REP assay has subsequently been adapted for use with other enteroviruses [21] demonstrating its versatility as a tool for the study of recombination.

In this study, we have utilised the CRE-REP assay to investigate the capacity for inter-species recombination within the coding region. Using assays for four different enterovirus species—A to D—we show inter-species recombination occurs readily at the VP1/2A (P1/P2) gene boundary. However, we found that the generation of recombinant genomes was not reflected in the production of infectious progeny virus. Using a constructed cDNA of one of the inter-species recombinants identified, we then demonstrated that the inability to yield progeny virus was due to a restriction in de novo positive-sense RNA synthesis. We propose that recombination can occur freely at the genome level and is not bound by species classification, but the production of a viable virus is restricted by the incompatibility of one or more components of the virus replication cycle.

## 2. Materials and Methods

### 2.1. Cell Culture

MRC-5 (human foetal lung fibroblast: ATCC CCL-171) and H1299 (human non-small lung carcinoma: CRL-5803) cells were maintained at 37 °C, 5% CO_2_, in Dulbecco’s modified Eagle medium (Fisher Scientific, Loughborough, UK; DMEM) supplemented with 10% heat-inactivated FBS (Fisher Scientific, Loughborough, UK; FBS-DMEM).

### 2.2. CRE-REP Assay, Plasmids, In Vitro RNA Transcription, and Transfection

The CRE-REP assay used to generate recombinant viruses has been described previously [16] and was repeated here. De novo synthesised acceptor templates EV71/CRE and EV68/CRE were generated using standard molecular biology techniques. For EV71/CRE, mutations to disrupt the 2C CRE were introduced into pT7-EV71 [22] and confirmed by sequencing. For EV68/CRE, mutations were introduced into the 2C CRE terminal loop of pT7-EV68 and confirmed by sequencing. The conserved AAA triplet has been demonstrated as the primary template for VPg uridylylation [23] and mutations here proved sufficient to block positive-strand RNA synthesis. EV68/PV1-1 was constructed via a 2-step overlapping PCR strategy using cDNAs for EV-D68 and PV1/rep as templates. Briefly, nt 1 to 3642 of EV-D68 was amplified with primers EV68_1(F) and EV68_3642(R) (Table S2) to include a 5′ SmaI site and a 3′ PV1/rep overlap of nt 2334 to 2353, and nt 2334 to 5105 of PV1/rep was amplified with primers PV1_2334 (F) and PV1_5105(R) (Table S2). The two PCR products were joined via a further round of PCR amplification using primers EV68_3642(F) and PV1_5105(R), digested with SmaI and BstBI and ligated into similarly digested PV1/rep. All PCR reactions were amplified using *Pfu* polymerase (Promega, Southampton, UK) with an initial denaturing at 95 °C for 1 min, followed by 30 cycles of 95 °C for 30 s, 50 °C for 30 s and 72 °C for 2 min/kb, and a final extension at 72 °C for 10 min.

Plasmids were linearised at the 3′ end of the virus sequence and RNA transcribed using a HiScribe T7 High Yield RNA Synthesis Kit (NEB, Hitchin, UK), following the manufacturer’s protocol. RNA transcripts were DNaseI (NEB, Hitchin, UK) treated to remove residual template DNA and column purified using a GeneJET RNA Purification Kit (ThermoFisher, Waltham, MA, USA) prior to spectrophotometric quantification. For all CRE-REP assays, equimolar amounts of both template RNAs (based on 250 ng of acceptor) were prepared with Lipofectamine 2000 (Fisher Scientific, Loughborough, UK) in a 3:1 Lipofectamine 2000:RNA ratio as per manufacturers’ protocol and transfected into 80% confluent MRC-5 or H1299 cell monolayers. The supernatant was recovered at 72 h post-transfection for virus quantification by plaque assay on MRC-5 cells, or TCID_50_ on H1299 cells.

### 2.3. RNA Extraction and RT-PCR

Total RNA was isolated from cells using a GeneJET RNA Purification Kit (ThermoFisher, Waltham, MA, USA) following 3 cycles of freeze–thawing and clarification by centrifugation at 10,000× *g* for 3 min. RNA was reverse transcribed at 42 °C using oligo dT, or EV68 specific primer (EV68(-)), and SuperScript II reverse transcriptase (Fisher Scientific, Loughborough, UK) as per manufacturers’ protocol. The region of recombination (VP1 to 2C) was amplified using virus-specific primers (Table S1) and Taq polymerase (NEB, Hitchin, UK) with an initial denaturing at 95 °C for 30 s, followed by 35 cycles of 95 °C for 30 s, 50 °C for 30 s and 68 °C for 1.5 min, and a final extension at 68 °C for 5 min. 

### 2.4. Sanger Sequencing Analysis

PCR products of recombinants from CRE-REP assays were analysed by Sanger sequencing (Eurofins Genomics, Cologne, Germany), and the recombination junctions were determined by aligning against parental reference sequences using SnapGene software v4.0.8.

### 2.5. PCR Cloning

PCR products were cloned using the pGEM-T Easy Vector System (Promega, Southampton, UK) as per manufacturers’ instructions, with positive colonies selected by blue/white screening and sequenced with M13 forward and reverse primers.

## 3. Results

### 3.1. Inter-Species CRE-REP Assays

**Figure 1:**
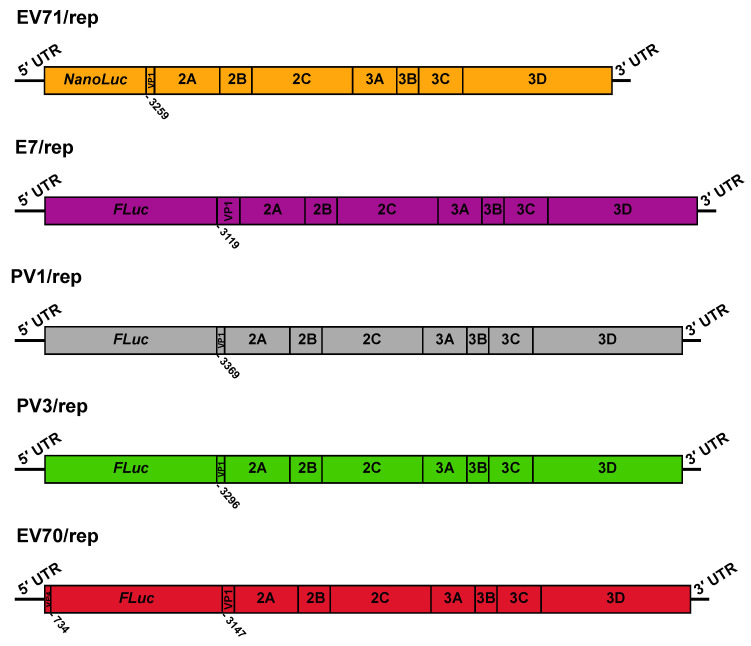
Schematic representation of donor replicon RNA templates. The genetic structure is shown for EV71/rep, E7/rep, PV1/rep, PV3/rep, and EV70/rep. Numbers below refer to the nucleotide number within VP1 at which the virus sequence restarts, relative to its position in the full-length genome. For EV70/rep, we also denoted the last nucleotide of the VP4 fragment present upstream of the luciferase gene.

**Figure 2:**
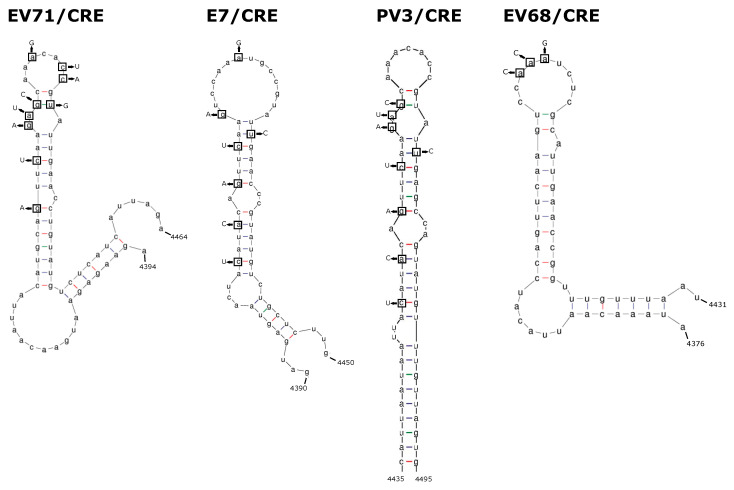
Representations of 2C-CRE stem-loop structures and mutations. The wild-type structure is shown for the 2C CRE of EV-A71, E7, PV3, and EV-D68. Nucleotides in boxes denote those mutated to knockout the CRE function, with replacement nucleotides shown with arrows.

The extreme rarity of inter-species recombinants isolated in nature suggests that recombination outside of the 5′ UTR is severely restricted between viruses of different species. We sought to formally test this theory and investigate the potential for inter-species recombination using CRE-REP assays adapted for multiple enteroviruses. We utilised a range of available replicon donor and CRE mutant acceptor templates covering enterovirus species A–D, while CRE mutants representing species A and species D were constructed de novo for this study (Figure 1 and Figure 2; Table 1; Section 2). The nomenclature was simplified from published forms and standardised across all templates; donor replicons for EV-A71 (species A), E7 (species B), PV1 and PV3 (species C), and EV-D70 (species D) were given the ~/rep suffix, with acceptor templates for EV-A71 (species A), E7 (species B), PV3 (species C), and EV-D68 (species D) given the suffix ~/CRE (Table 1).

### 3.2. Isolation of Recombinants

With the five donor and four acceptor templates available, a total of five intra-species control assays, and fifteen inter-species CRE-REP assays could be performed. Of the enteroviruses selected for this study, wild-type EV-D68 and EV-D70 have an optimal growth temperature of 33 °C, compared with 37 °C for all other selected viruses. In order to provide the maximal conditions for the growth of potential recombinant viruses, CRE-REP assays were carried out at both 33 °C and 37 °C, and in two cell lines—MRC-5 and H1299 cells—previously tested and demonstrated to support the full replication cycle of each of the wild-type enteroviruses. As expected from previous studies demonstrating that CRE mutations do not revert [16], transfection of individual /CRE or /rep genomes failed to generate infectious progeny virus when assessed by plaque assay or TCID_50_ assay on fresh MRC-5 or H1299 cells, respectively. Cells were subsequently co-transfected with equimolar amounts of template RNAs, in duplicate, and incubated for 72 h prior to harvest of cellular supernatant and the presence of infectious recombinant progeny virus was determined by assay, as above.

Infectious progeny virus was observed only for intra-species co-transfections of species A, B, and C, with no infectious progeny viruses generated from the species D intra-species combination of EV68/CRE + EV70/rep following three independent tests (Table S1). Similarly, from 3 test attempts, none of the 15 tested inter-species assays generated infectious extracellular progeny virus, as determined by plaque or TCID_50_ assay. The absence of infectious progeny was supported by the inability to amplify recombinant sequences from purified extracellular RNA using RT-PCR and virus-specific primers that target the P1/P2 junction (Table S2).

**Figure 3:**
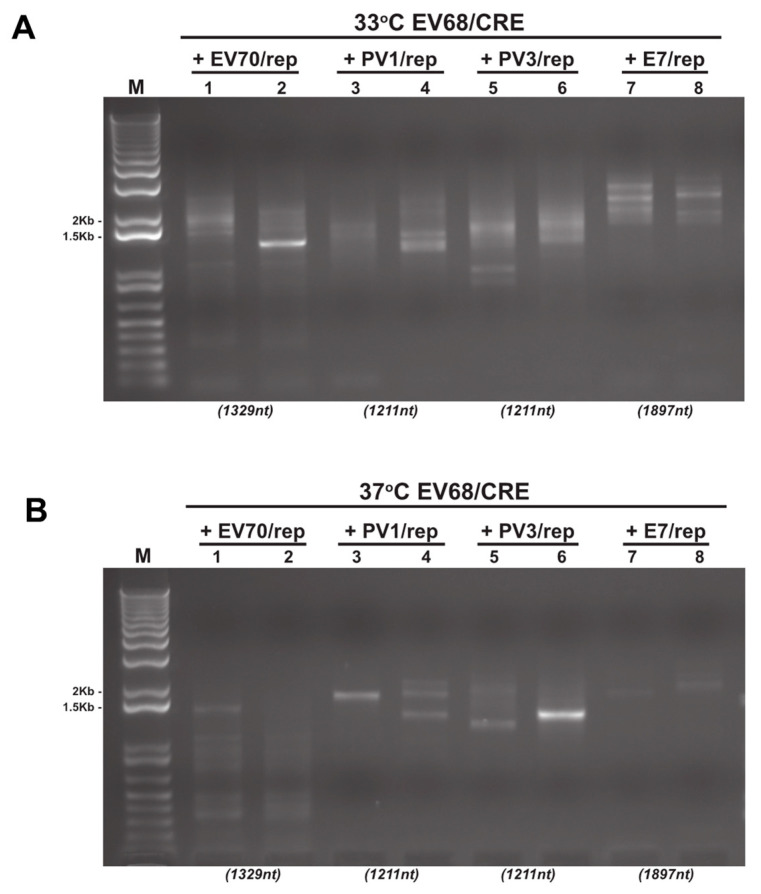
Reverse-transcription PCR analysis of CRE-REP assay samples. Example gel electrophoresis analysis of total RNA isolated and amplified by RT-PCR from CRE-REP assays. In this example, we show results from both 33 °C (**A**) and 37 °C (**B**) following co-transfection of the EV68/CRE acceptor template with either a species B, C, or D donor template. Lanes are numbered 1–8 for reference. Numbers in brackets below gel images are the expected sizes for a precise recombinant of each assay template pairing.

Recently, we have demonstrated that the polymerase strand transfer event of recombination is a recurring, promiscuous process, generating a much greater range of recombinant genomes than is represented in the infectious virus population [5,27]. We, therefore, hypothesised that inter-species recombinant RNAs may still be being generated but are unable to produce infectious progeny virus. To investigate this possibility, we repeated all the CRE-REP assays as above but instead harvested total cellular RNA at 8 h post-transfection. As exemplified in Figure 3, RT-PCR analysis of all samples now revealed a variety of PCR products for each assay. These products differed between temperatures and cell lines but also between duplicate samples of individual assays (Figure 3 and additional gel analyses not shown).

Depending on the assay, and therefore the primer pair used, precise genome-length PCR products (i.e., lacking insertions or deletions at the junction [16]) of potential recombinants were within the range ~1.2 to ~2.5 Kb. Only a small number of samples yielded a single visible product of approximately the expected size (e.g., Figure 3B; lane 6). In contrast, the majority exhibited a diversity of PCR product sizes consistent with previous observations and the generation of diverse populations of recombinant genomes [5,12,16,28,29,30]. Of these products, many were considerably smaller than the size expected for a precise genome-length recombinant (e.g., Figure 3A,B; lane 1), though some were substantially larger. These results indicate the presence of a recombinant RNA population in both the type D intra-species assay (EV70/rep + EV68/CRE; Figure 3A,B; lanes 1 and 2), as well as a variety of inter-species recombinant RNA populations (Figure 3A,B; lanes 3–8).

### 3.3. Intra-Species EV68/70 Recombinant Genome Analysis

To further investigate the potential recombinant RNA populations, we first focused on the species D intra-species assay of EV70/rep + EV68/CRE, which did not generate a viable virus. Although there is only approximately 75% nucleotide sequence identity between EV-D70 and EV-D68, this is not dissimilar to the ~80% identity between PV1 and PV3 which recombine freely [8].

**Figure 4:**
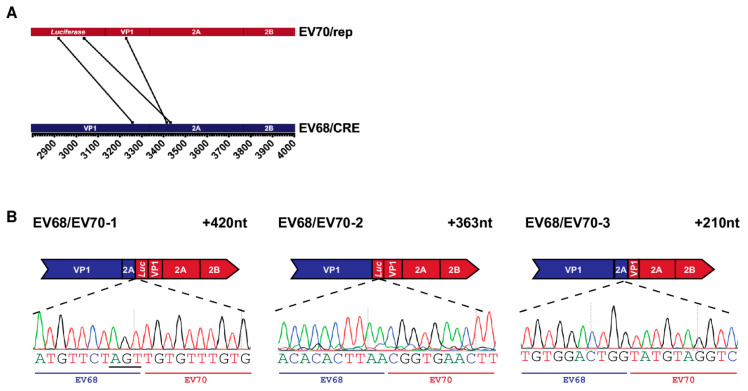
Recombinant genomes isolated from EV68/EV70 CRE-REP assay: (**A**) parallel coordinates visualisation of EV68/EV70 recombinant genomes. The location of each recombination junction was mapped respectively to each parental genome. Each line represents a unique recombinant; (**B**) schematic representation of each identified recombinant. Recombinant name is given top left and the size of each sequence duplication is top right. The relevant part of the recombinant sequence chromatogram is shown below, with the acceptor and donor-derived regions indicated based on the determined junction position. Nucleotides underlined in recombinant sequence represent regions of ambiguity where both parental sequences match. Although labelled as acceptor, it cannot be determined from which template these particular nucleotides were derived.

To analyse potential recombinant RNAs, we utilised PCR cloning to isolate products following RT-PCR. Due to the variety of products amplified for some samples, and the presence of multiple low-molecular-weight products that would impact the range of DNA fragments cloned, we limited our analysis to samples showing more prominent bands at the approximate expected size for precise, genome-length recombinants which, in the case of the primer pair used for EV-D70 and EV-D68, was 1.3 Kb. Entire PCR reactions, amplified from MRC-5 cells at 33 °C, transfected with EV70/rep and EV68/CRE RNA, were TA cloned, blue/white screened for positive colonies, and subject to repeat PCR amplification of the recombination region. Products under 500 bp were excluded from further analysis, as these were deemed unlikely to correspond to viable recombinants. Sanger sequencing of a small number of positive clones identified the presence of three unique recombinant sequences between EV-D68 and EV-D70 (Figure 4A; Table 2).

The genomes of all three recombinants had imprecise junctions spanning the VP1/2A boundary and contained insertions of 210, 363, and 420 nt, respectively, meaning that all genomes were in-frame and therefore, theoretically, replication competent. The junction of the first identified recombinant, EV68/EV70-1, occurred at a short region—3 nt—of complete sequence identity between the parental RNA templates, resulting in an ambiguous junction in which it was not possible to determine the exact position at which crossover occurred (Figure 4B, underlined nts). While the sequence insertions in EV68/EV70-1 and -2 are at the higher end of the size range of insertions observed in our poliovirus studies, we have previously isolated infectious recombinants with insertions of up to 411 nt in size [5], the related foot-and-mouth disease virus genome is ~8.2 kb [31], and artificially constructed poliovirus genomes have been shown to accommodate sequence inserts of up to 573 nt [32]. It was therefore unlikely that either genome size restriction or the presence of duplicated sequence could explain the lack of infectious virus isolated from the assay. Further studies will be needed to determine where the block occurs in the formation of EV68/EV70 recombinants and whether there is a commonality with the observed failure of some species C to also undergo intra-species recombination [33].

### 3.4. Inter-Species Recombinant Genome Analysis

**Figure 5:**
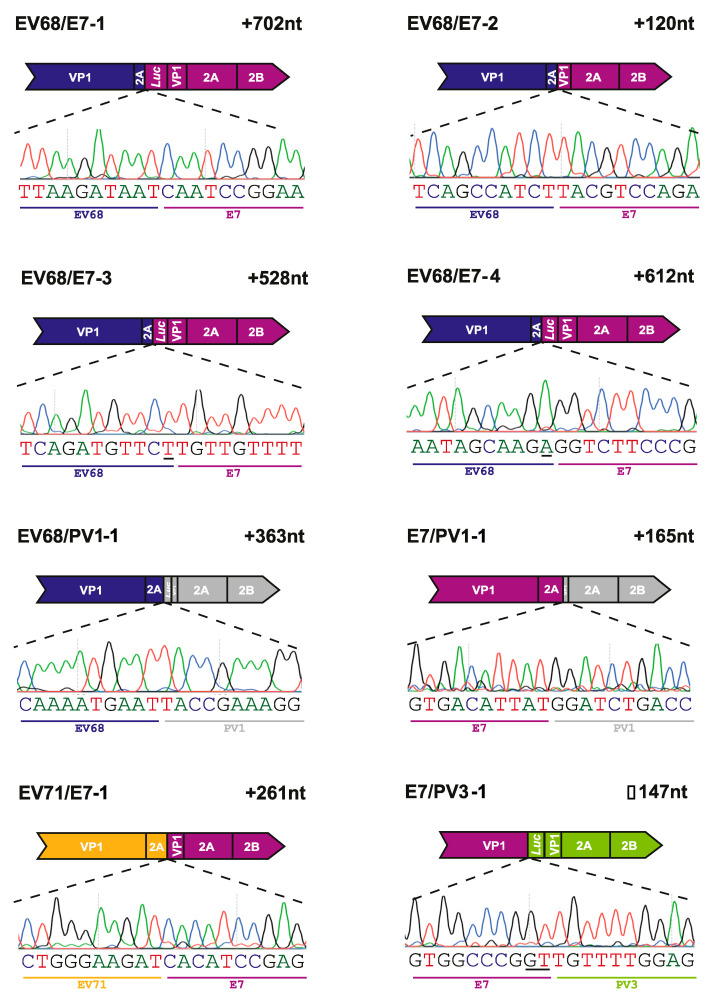
Recombinants isolated from inter-species CRE-REP assays. Schematic representation of each identified recombinant. Labelling and presentation are as described in Figure 4B.

Following the analysis of the EV68/EV70 RNA population and the identification of recombinant genomes, we applied the same process to the inter-species CRE-REP assays. As with the EV68/EV70 assay, the inter-species RT-PCRs had yielded variable results. As previously described in Section 3.3, we, therefore, limited which assays would be subject to whole PCR cloning based on the observation of prominent gel electrophoresis bands at the approximate sizes for precise recombinant genomes. Five assays were selected for analysis across the spectrum of possible species crosses: EV71/E7 (species A + B), E7/PV1 (species B + C), E7/PV3 (species B + C), EV68/E7 (species D + B) and EV68/PV1 (species D + C), and a total of eight unique inter-species recombinant genomes were identified following Sanger sequencing (Figure 5; Table 2).

All eight recombinant genomes identified were imprecise, with junctions spanning the VP1/2A boundary, as observed in the EV68/EV70 assay. Seven of the recombinants contained an in-frame insertion, with size duplications ranging from 120 to 702 nt. Of these, three—EV68/E7-1, EV68/E7-3, and EV68/E7-4—contained insertions over 500 nt with duplications of 702 nt, 528 nt, and 612 nt, respectively. One recombinant genome, isolated from the E7/PV3 assay, contained an in-frame deletion of 147 nt when compared with the length expected for a recombinant with a precise junction. The nature of the polymerase strand transfer event in this recombinant resulted in a considerable deletion of the VP1 sequence. Although this recombinant was in-frame, the deletion of the VP1 sequence alone would explain why this genome was unable to produce infectious progeny.

### 3.5. Inter-Species Recombinant EV68/PV1-1 Genome Generates Negative-Strand RNA

**Figure 6:**
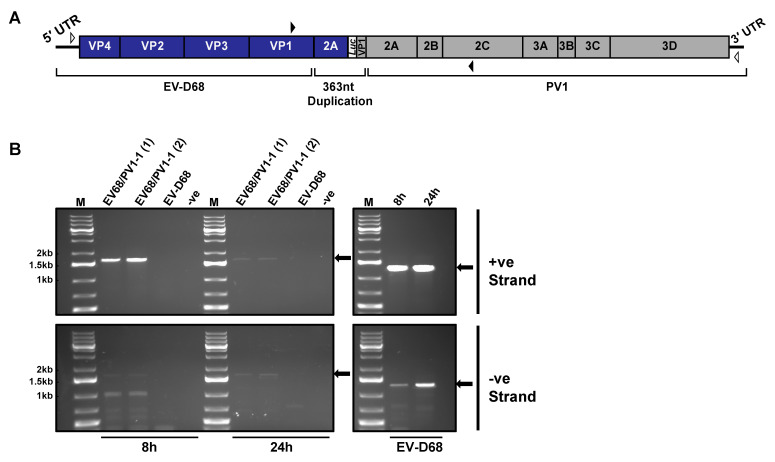
Replication of inter-species recombinant EV68/PV1-1: (**A**) schematic of inter-species recombinant genome EV68/PV1-1. Open arrowheads mark positions of primers for cDNA synthesis and closed arrowheads mark positions of primers for PCR; (**B**) PCR assay for negative- and positive-strand RNA. Left-hand panels denote amplification of recombination window with primers specific to the recombinant (EV68(F) and PV1(R)), and right-hand panels denote amplification of EV-D68 controls with EV-D68 specific primers (EV68(F) and EV68(R)). Amplification from positive-strand (upper panels) and negative-strand (lower panels) RNA is shown. Arrows indicate expected product sizes.

Although our screen identified a number of inter-species recombinant genomes, the inability to isolate infectious virus meant that questions remained as to whether these genomes were replication competent but could not be packaged or were simply dead-end products. To test this, we constructed a cDNA for one of the recombinant genomes, EV68/PV1-1 (Figure 6A). Confirming our previous lack of success in recovering viable virus from recombination between these parental genomes, we were unable to isolate infectious virus from cellular supernatant following transfection of MRC-5 cells. To determine if the genome was capable of replicating, we then transfected MRC-5 cells in duplicate with 250 ng of EV68/PV1-1 RNA and harvested total RNA at both 8 and 24 h post-transfection. Wild-type EV-D68 was transfected alongside as a positive control. Although the optimal temperature for growth of EV-D68 is 33 °C, all transfections were incubated at 37 °C to match the conditions of the original CRE-REP assay from which EV68/PV1-1 was isolated. We then conducted RT-PCR assays for both positive- and negative-strand RNAs from purified total cellular RNA, utilising primers specific for either EV68/PV1-1 or wild-type EV-D68 to generate products of 1574 nt or 1278 nt, respectively (Figure 6B; Table S2). We could not detect positive- or negative-strand RNA for EV-D68 with the EV68/PV1-1 specific primers, confirming that we were correctly detecting the recombinant EV68/PV1-1.

To aid analysis, all gel electrophoresis products were quantified using ImageJ (Table S3). The EV-D68 positive control showed the presence of both positive- and negative-strand RNA at 8 h, with levels increasing by 3% and 26%, respectively, at 24 h, as would be expected for actively replicating RNA (Figure 6B; right-hand panels). In contrast, for replicates of EV68/PV1-1, we observed a 20–30% decrease in levels of positive-strand RNA between 8 and 24 h (Figure 6B; top left panel), suggesting that positive-strand synthesis is severely compromised. As newly replicated positive-strand RNAs are indistinguishable from input RNA, it is also possible that at 24 h, we were also still detecting some residual input RNA. EV68/PV1-1 negative-strand RNAs were detected at low levels at 8 h, together with a number of lower molecular weight products which may represent either non-specific priming or a range of aberrant products generated during negative-strand synthesis. At 24 h, the negative-strand replicates showed similar increases of 16–24% over time compared with the control, EV-D68, with significantly decreased levels of lower molecular weight products now observed.

When the level of negative-strand RNA was determined as a ratio of total RNA, we observed a small increase from 35% to 39% between 8 h and 24 h for the control, EV-D68. In comparison, the ratio of negative- to positive-strand for both EV68/PV1-1 replicates increased from 35–36% to 47%. The observation of an increased ratio of negative- to positive-strand RNA again suggests that it is a restriction on positive-strand RNA synthesis that contributes to the lack of isolation of infectious virions.

Overall, these results suggest that at least some inter-species recombinants are replication competent with respect to negative-strand synthesis, a finding that may have a considerable impact on the potential for the evolution of these viruses.

## 4. Discussion

Recombination is a known driver of evolution among viruses with single-stranded, positive-sense RNA genomes. The exchange of large regions of genetic material via recombination can have dramatic effects on fundamental biological functions, including altering replication kinetics, pathogenicity, and cell or host tropism. Such changes are most notable when resulting in the emergence of novel viruses that increase the threat to animal or human health such as Western Equine Encephalitis virus [34], circulating vaccine-derived polioviruses [9,10], SARS coronavirus [35,36], and likely SARS-CoV-2, the causative agent of the COVID19 pandemic [37,38].

Using PV as a model system, we have recently shown that intra-species recombination—i.e., between two viruses of the same species—is a ubiquitous event, leading to the generation of a wide range of recombinant genomes from which only a select number of infectious progeny viruses are isolated [5,27]. In the current study, we utilised an in vitro recombination assay known as the CRE-REP assay [16], to investigate the potential for inter-species recombination.

Focusing on the human enteroviruses, comprising species A–D, we developed a number of CRE-REP assays that would allow for the isolation of inter-species recombinants (Figure 1 and Figure 2). Despite repeated attempts, we were unable to isolate infectious progeny virus from inter-species assays under any condition, as demonstrated by the absence of detectable supernatant virus via plaque, TCID50, or RT-PCR assay. While this result fits with the lack of inter-species enterovirus recombinants isolated in the field, it remains a possibility that recombinant viruses had significantly different growth requirements to the parental genomes that were not met by the conditions under which we tested. It is also possible that recombination between pairs of enteroviruses other than those tested here could yield infectious progeny. However, with over one hundred members within species A-D alone, it is not feasible within this proof-of-concept study to test all combinations in order to confirm which, if any, are capable of producing viable recombinants.

We were also unable to isolate infectious progeny from the EV68/EV70 intra-species CRE-REP assay, indicating that more diverse intra-species pairings may also be unable to generate viable recombinant viruses, as shown by Bessaud et al. [39] when demonstrating the requirements for viable recombinants between PV2 and EV-C99. A similar observation was made by Liu et al. in a study of recombination between the species C enteroviruses, PV, and coxsackievirus A20 (CVA20) [33]. It was shown that although chimeric genomes of PV and CVA20 could be translated and were replication competent, encapsidation was blocked preventing the release of infectious progeny virus. This was found to be the result of direct interaction between the 2C^ATPase^ domain of PV and VP3 of CVA20, and encapsidation could be rescued if these two regions originated from the same virus [33]. We investigated whether this interaction could explain the inability to generate infectious recombinant virus in the EV68/EV70 assay by replacing the 2C sequence of EV70/rep—the EV-D70 donor replicon—with that of EV-D68, such that any recombinants generated during the CRE-REP assay would now contain both VP3 and 2C regions originating from EV-D68. However, we were still unable to isolate any infectious recombinant viruses (data not shown), indicating that restoring the VP3/2C interaction does not, at least in this instance, restore the replication defects observed by Liu et al. It is quite likely that there is more than one protein–protein or protein–RNA interaction that is key to the production of viable recombinants, and these essential interactions may differ between enterovirus species.

Our previous research has shown that in both CRE-REP assays and co-infection models, there is a marked difference in the number of infectious recombinant viruses isolated, compared with the number of recombinant RNA genomes that can be detected [5,27]. We, therefore, sought to establish whether inter-species recombinant genomes were being generated but were unable to yield infectious viruses. Using RT-PCR analysis of total cellular RNA at 8 h post-transfection, we were able to detect genomes relating to eight inter-species recombinant genomes (Figure 3 and Figure 5), as well as three intra-species genomes from the EV68/EV70 CRE-REP assay (Figure 3 and Figure 4). All but one of the recombinant genomes—EV7/PV1-1—were isolated from assays carried out in MRC-5 cells, and all but two—EV7/PV1-1 and EV68/PV1-1—were isolated at 33 °C. While these numbers are likely biased by the samples that were selected for analysis (reflecting our cloning strategy), overall, the majority of samples from H1299 cells yielded poor amplification of products, explaining why all but one recombinant were isolated from MRC-5 cells. Similarly, more products were amplified and analysed from samples at 33 °C, the optimal temperature for EV-D68, perhaps explaining why more recombinants were isolated with an EV-D68 backbone. Furthermore, links between temperature and recombination have been previously demonstrated [40], and it may be possible to exploit such processes in future studies—for example, through the use of cold-adapted strains of poliovirus—to increase the range of markers suitable for the selection and isolation of inter-species recombinants.

There are a number of reasons why viable viruses may not be produced from the recombinant genomes detected in this study including restrictions in nucleic acid synthesis (positive or negative strand), polyprotein processing defects, or an inability to undergo packaging. We investigated one of these potential reasons and found that for the species D/C recombinant EV68/PV1-1 (Figure 6A), positive-strand but not negative-strand RNA synthesis was disrupted (Figure 6B). To have been identified in the initial screen, EV68/PV1-1 must be capable of generating positive-strand genomes to a level sufficient for detection via RT-PCR. However, as observed, this ability is significantly reduced when compared with the replication of wild-type EV-D68 (Figure 6B). It is possible that replication of the negative-strand RNA is also inefficient, therefore contributing to the overall reduced RNA levels. While we have demonstrated one potential defect, further in-depth studies utilising EV68/PV1-1, as well as additional interspecies recombinants, will be required to fully assess genome replication deficiencies. It is known that productive PV replication, including initiation of positive-strand RNA synthesis, requires a complex of RNA–RNA and RNA–protein interactions involving the viral 5′ UTR cloverleaf structure and stem-loop IV, and the binding of host protein PCBP and viral protein 3CD [41,42,43]. If the binding of 3CD to the cloverleaf structure is altered due to the protein and RNA now being derived from two separate viruses in the recombinant, this disruption may impact RNA synthesis. However, a number of studies have successfully swapped the 5′ UTRs of different enterovirus species, suggesting that this interaction is quite flexible in terms of binding partners. While studies swapping the 5′ UTR of PV with human rhinovirus 14 (HRV-14) abrogated RNA replication [14,44], others have identified viable recombinants between different enterovirus species, although replication was impaired depending on which group the 5′ UTR belonged to [12]. These varying observations may suggest that more than one interaction is disrupted by the chimeric nature of inter-species recombinants.

Our analysis of inter-species enterovirus recombination was not intended to be exhaustive but to instead investigate the presence and basic features of any recombinant genomes generated. We have clearly demonstrated that recombinant genomes with crossovers within the coding region are generated even though infectious progeny virus is absent. Based on the data available, including the analysis of EV68/PV1-1, it is likely that interspecies recombinant genomes are, at least partially, replication competent. Firstly, it was notable that all recombinant genomes were located at the VP1/2A boundary separating the P1 and P2/3 ‘modules’ that encode the structural and non-structural proteins, respectively. This is compared with our previous studies in which we found 55–70% of junctions spanned the VP1/2A boundary with the remainder located at the 2A/2B gene boundary [5,16,27] and is perhaps suggestive that any subsequent functionality requires these modules to be largely uninterrupted. Using a modified poliovirus CRE-REP assay, in which the functional CRE of the donor template was relocated to the 3′ UTR, we have previously demonstrated that recombination events occur throughout the coding region. The use of similarly modified inter-species templates may be valuable in further studies to help delineate the true functional boundaries for genome replication. Secondly, all genomes isolated, both with insertions and deletions, were in-frame genomes. We have previously shown that in-frame recombinant genomes are selected over time via repeated replicative recombination events [27], and it is likely that the genomes isolated in this study would be subject to the same fitness selection pressures. If these selection pressures are sufficient to maintain functional genomes, then previous evidence would suggest a process of continual evolution until a cell’s resources are depleted, or the genome acquires the ability to produce infectious progeny and propagates further. Deep-sequencing studies, similar to those we have previously conducted [5,27], could provide larger data sets of recombinant genomes and, in combination with mutagenesis studies and further in vitro analysis, help shed light on the determinants needed for the generation of replication-competent genomes.

The isolation of recombinants with junctions at the boundary between the structural and non-structural modules suggests there may be a risk of generating novel viruses with altered tropism, as was determined for SARS coronavirus [35,36]. Of course, with the small genomes of the enteroviruses, the semantics of whether these recombinants are truly novel viruses remain to be determined. If fully replication competent, do they exhibit the combined characteristics of the two parental genomes, or are they simply pre-existing capsid-coding modules propagated by a newly combined replication module? In this regard, it is notable that, whilst the capsid is known to account for much of the cell or host tropism determinants, there are features within the non-structural proteins that may also contribute to the phenotype of the virus, including drug resistance [45] and polymerase fidelity [46]. Although repeated attempts to isolate infectious progeny were not successful, it has been shown previously that repeated passaging can lead to mutations capable of restoring interactions that are otherwise inhibited in recombinant virus genomes [33]. Larger scale studies to understand more about the restriction to genome replication in these types of recombinants may help in predicting the potential and likelihood of generating novel viruses with altered characteristics.

## Figures and Tables

**Table 1:** Acceptor and donor templates.

Template Type	Name	Virus	Species	Reference
**Donor**	EV71/rep	EV-A71	A	Tee et al. (2016) [24].
	E7/rep	E7	B	Lowry (2011) [25].
	PV1/rep	PV1	C	Lowry et al. (2014) [16].
	PV3/rep	PV3	C	Lowry et al. (2014) [16].
	EV70/rep	EV-D70	D	Waugh (2007) [26].
**Acceptor**	EV71/CRE	EV-A71	A	Tan et al. (2016) [22] and this paper (see Section 2).
	E7/CRE	E7	B	Lowry (2011) [25].
	PV3/CRE	PV3	C	Lowry et al. (2014) [16].
	EV68/CRE	EV-D68	D	This paper (see M&M).

**Table 2:** Recombinant genomes.

Recombinant	Cell Line	Temp (°C)	5′ nt ^a^	3′ nt ^b^	Imprecise	Ambiguity
EV68/EV70-1	MRC-5	33	3432	2277	+420	3
EV68/EV70-2	MRC-5	33	3258	2160	+363	0
EV68/EV70-3	MRC-5	33	3415	2470	+210	0
EV68/E7-1	MRC-5	33	3360	1954	+702	0
EV68/E7-2	MRC-5	33	3404	2580	+120	0
EV68/E7-3	MRC-5	33	3429	2197	+528	1
EV68/E7-4	MRC-5	33	3472	2156	+612	1
E7/PV1-1	H1299	37	3840	2425	+165	0
EV68/PV1-1	MRC-5	37	3572	2334	+363	0
EV71/E7-1	MRC-5	33	3438	2456	+261	0
E7/PV3-1	MRC-5	33	2904	2220	−147	2

^a^ The 5′-most nucleotides matching to the acceptor template; ^b^ the first nucleotide then matching to the donor template.

## Data Availability

The data presented in this study are available in the manuscript and supplementary data.

## Supplementary Materials

The following are available online at https://www.mdpi.com/article/10.3390/v13122390/s1, Figure S1: CRE-REP assay, Table S1: CRE-REP assays and isolations, Table S2: Primer sequences, Table S3: Quantitative analysis of EV68/PV1-1 PCR.
