# Supplementary material for: Isolation and Identification of Inter-Species Enterovirus Recombinant Genomes
Source: Viruses. 2021 Nov 29;13(12):2390. doi: 10.3390/v13122390 (PMC8703282; doi:10.3390/v13122390)
Supplement: Supplementary file 1 [file viruses-13-02390-s001.zip › Figure S1.pdf]

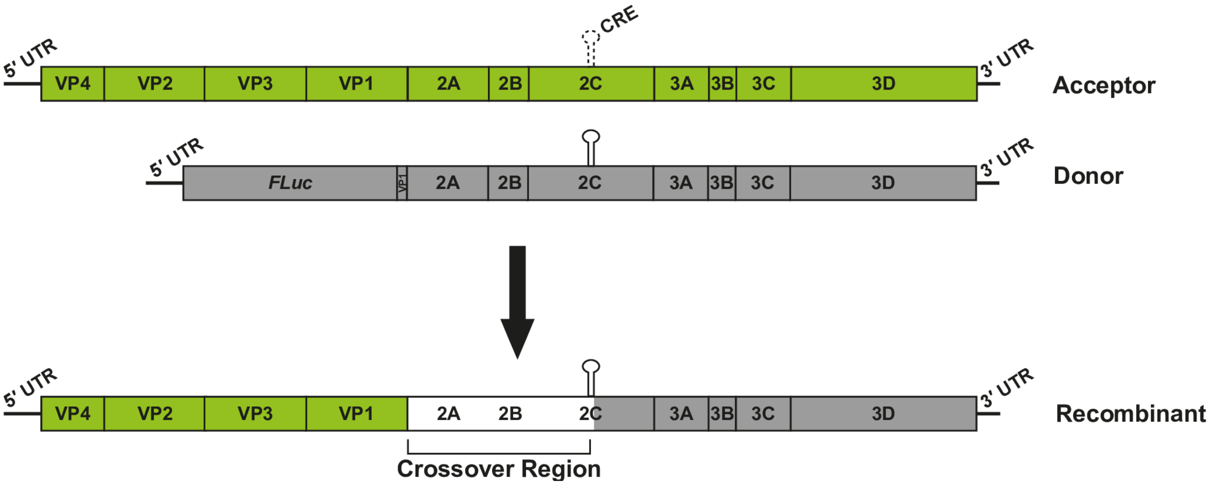

Figure S1. CRE-REP assay. A full-length acceptor RNA template containing mutations to the 2C CRE, which inhibit positive-strand synthesis, is co-transfected into permissive cells with a donor RNA template in which the structural genes have been replaced with a luciferase reporter gene. If recombination occurs within the crossover region viable recombinant viruses can be generated and isolated.
