# Supplementary material for: Isolation and Identification of Inter-Species Enterovirus Recombinant Genomes
Source: Viruses. 2021 Nov 29;13(12):2390. doi: 10.3390/v13122390 (PMC8703282; doi:10.3390/v13122390)
Supplement: Supplementary file 1 [file viruses-13-02390-s001.zip › Table S1_Assays.pdf]

Table S1. CRE-REP assays and isolations

Donor	Acceptor	Recombinant Genomes[a]	Recombinant Virus
EV71/rep	EV71/CRE		X
	E7/CRE		
	PV3/CRE		
	EV68/CRE		
E7/rep	EV71/CRE	X	
	E7/CRE		X
	PV3/CRE		
	EV68/CRE	X	
PV1/rep	EV71/CRE		
	E7/CRE	X	
	PV3/CRE		X
	EV68/CRE	X	
PV3/rep	EV71/CRE		
	E7/CRE	X	
	PV3/CRE		X
	EV68/CRE		
EV70/rep	EV71/CRE		
	E7/CRE		
	PV3/CRE		
	EV68/CRE	X	

[a] as determined by PCR analysis
