# Supplementary material for: Isolation and Identification of Inter-Species Enterovirus Recombinant Genomes
Source: Viruses. 2021 Nov 29;13(12):2390. doi: 10.3390/v13122390 (PMC8703282; doi:10.3390/v13122390)
Supplement: Supplementary file 1 [file viruses-13-02390-s001.zip › Table_S2_Primers.pdf]

Table S2. Primer Sequences

PCR	Primer	Sequence (5′-3′)
Recombinant amplification	EV71(F)	GTGCGAACTGTGGGTTCATC
	EV71(R)	GTGGTACTTGTCTGCTATGGC
	E7(F)	CAAGCTGTACGCACGTCATG
	E7(R)	GTGTCTCTGGTGCAACACTG
	PV3(F)	GCAAACATCTTCCAACCCGTCC
	PV3(R)	TTGCTCTTGAACTGTATGTAATTATTAATGG
	PV1(R)	TTGCTCTTGAACTGTATGTAGTTG
	EV68(F)	ATGCCATACATGAGCATTGC
	EV68(R)	TGAGGCTACTGATTTGCCAG
	EV70(R)	ATCAGGTGGGAGTGAGTAAG
EV61/PV1-1 cloning	EV68_1(F)	TACGCTCCCGGGCTTTGTACGCCTGTTTTAATTCCCT
	EV68_3642(R)	TCCGGTAAGACCTTTCGGTACCACCACAATCTCCTGCTTC
	PV1_2334(F)	GAAGCAGGAGATTGTGGTGGTACCGAAAGGTCTTACCGGA
	PV1_5105(R)	TGGACGGGGCATTTATGATT
cDNA synthesis	Oligo dT – for all positive strand	TTTTTTTTTTTTTTT
	EV68(-) – for negative strand	ACGTGGCGGCTAGTACTC
