# Supplementary material for: Isolation and Identification of Inter-Species Enterovirus Recombinant Genomes
Source: Viruses. 2021 Nov 29;13(12):2390. doi: 10.3390/v13122390 (PMC8703282; doi:10.3390/v13122390)
Supplement: Supplementary file 1 [file viruses-13-02390-s001.zip › Table_S3.pdf]

Table S3. Quantitative analysis of EV68/PV1-1 PCR

Construct	RNA strand	Time point	Area	Mean	Min	Max	Integrated Density	Raw Integrated Density	% change from 8 to 24h	Negative-strand (% total RNA)
**EV68/PV1-1 Replicate 1**	Positive	8h	0.072	32941.286	20966	64724	2376.801	153341687		
		24h	0.072	26474.391	24536	31768	1910.197	123238289	-20%	
	Negative	8h	0.072	18783.735	16682	21734	1355.296	87438288		36%
		24h	0.072	23303.048	21338	27265	1681.377	108475689	24%	47%
**EV68/PV1-1 Replicate 2**	Positive	8h	0.072	37176.872	22405	64724	2682.41	173058339		
		24h	0.072	26149.449	24270	31356	1886.752	121725687	-30%	
	Negative	8h	0.072	20008.995	17821	24130	1443.702	93141874		35%
		24h	0.072	23301.075	20820	29442	1681.234	108466506	16%	47%
**EV-D68**	Positive	8h	0.072	43115.732	21907	64724	3110.914	200703733		
		24h	0.072	44269.077	18749	64724	3194.131	206072554	3%	
	Negative	8h	0.072	22454.473	18091	38862	1620.15	104525570		34%
		24h	0.072	28343.515	15661	64725	2045.06	131939063	26%	39%
